# The second decade of DTI in TBI part 1: a systematic review of mild TBI

**DOI:** 10.3389/fneur.2026.1734544

**Published:** 2026-04-10

**Authors:** Molly F. Charney, Simone Glajchen, Shawn Brain, Fahmida Rashid, Sabrina Kentis, Melvin Alexander, Arvind Dev, Jenasis Ortega, Chihiro Okada, Brian Morris, Timothy Darby, Taskin Forkan, Anthony D. Yao, Yuchen Dong, Cindy Zhou, Jane Wee, Emily Hunt, Caroline Delbourgo Patton, Michael L. Lipton

**Affiliations:** 1 Department of Neurology, Columbia University Irving Medical Center, New York, NY, United States; 2 Department of Radiology, Columbia University Irving Medical Center, New York, NY, United States; 3 Touro College of Osteopathic Medicine, New York, NY, United States; 4 Department of Radiology, University of California, Los Angeles, Los Angeles, CA, United States; 5 Albert Einstein College of Medicine, Bronx, NY, United States; 6 Department of Neurology, New York University Langone Hospital, New York, NY, United States; 7 Department of Radiology, Lennox Hill Hospital, New York, NY, United States; 8 Department of Radiology, Montefiore Medical Center, New York, NY, United States; 9 Albert Einstein College of Medicine, D. Samuel Gottesman Library, Bronx, NY, United States; 10 Department of Biomedical Engineering, Columbia University, New York, NY, United States

**Keywords:** cognition, DTI, neuroimaging, review, TBI

## Abstract

**Background:**

Traumatic brain injury (TBI) is a pervasive and important public health concern. TBI can range from mild, resulting in headache, dizziness, and imbalance, to severe resulting in coma and death. Diffusion tensor imaging (DTI) offers the ability to assess tissue microstructure at a level inaccessible to classical neuroimaging methods, such as CT and structural MRI. This systematic review aims to explore studies using DTI in mild TBI (mTBI) during the 2012–2022 decade, which is the second decade of reported use. The use of DTI in moderate-severe TBI (msTBI) during this time period is discussed in our companion systematic review.

**Methods:**

A systematic literature review was conducted in accordance with the Preferred Reporting Items for Systematic Reviews and Meta-Analyses (PRISMA) guidelines. We searched the electronic databases PubMed/MEDLINE, Embase, Cochrane Library, and Web of Science from 2012 through September 28, 2022.

**Results:**

A total of 325 studies on mild TBI were included, which encompassed 26,287 participants. There were more longitudinal studies in 2012–2022 compared to the prior decade (29.85 vs. 13%). Fractional anisotropy (FA) and mean diffusivity (MD) were the most commonly used DTI measures. Regardless of acquisition techniques and analysis methods, the majority of studies that compared FA between those with mTBI and controls, found lower FA in mTBI patients compared to controls, but less consistently than in msTBI. Lower FA was associated with worse cognitive outcomes across domains, but associations with clinical post-concussive symptoms were more mixed.

**Conclusion:**

Since its first decade (2002–2012) of reported use, DTI applications to mTBI have continued to expand in both quantity and scope, including notable increases in the proportions of larger and longitudinal studies, those employing whole brain analyses and those addressing clinical and cognitive outcomes. The most salient feature of the study results remains that low FA is the most common finding identified in mTBI patients compared to controls, however the direction of the FA effect is more variable for mTBI compared to msTBI.

**Systematic Review Registration:**

Prospero [CRD42022361318], https://www.crd.york.ac.uk/prospero/display_record.php?RecordID=361318.

## Introduction

It is estimated that over 42 million people worldwide experience mild traumatic brain injury (mTBI) annually (1). This figure is likely an underestimate given as many do not seek care for mild injuries. mTBI accounts for about 90% of all reported cases of traumatic brain injury (TBI). Prognosis varies significantly among those with mTBI. Many recover fully after an injury, but more than half of those of with mTBI go on to experience persistent symptoms after 6 months, including but not limited to headache, dizziness, and difficulties with balance (2). Persistent symptoms can be debilitating and significantly influence quality of life. mTBI can occur in the context of motor vehicle accidents, falls, assault including intimate partner violence, military service, and contact sports.

Neuroimaging with CT and conventional MRI after mTBI is by all diagnostic definitions non-revealing. However advanced neuroimaging techniques, such as diffusion tensor imaging (DTI), have demonstrated indications of underlying structural injury. The primary mechanism of tissue injury in mTBI is traumatic axonal injury (TAI) (3–5). DTI quantifies white matter microstructural injury and therefore is well-suited to detect TAI.

Since its first published application to TBI in 2002, DTI has been used extensively to characterize white matter effects of mTBI. A comprehensive systematic review reported on studies applying DTI to TBI from 2002–2011, its first decade of reported use (6). This systematic review encompasses published studies of DTI applied to mTBI during the next decade of use, 2012–2022. Due to the large number of studies published since 2011, we report on studies of moderate-severe TBI in a companion paper. We present here a comprehensive review of 325 studies, describe how this landscape has changed from the previous decade, compare mTBI findings to those reported in msTBI, and suggest implications for the field and future investigation.

## Materials and methods

### Protocol and registration

The protocol for this systematic review was registered in Prospero (CRD42022361318) and is available online (https://www.crd.york.ac.uk/prospero/display_record.php?RecordID=361318).

### Literature review

A systematic literature review was conducted by a medical librarian in accordance with the Preferred Reporting Items for Systematic Reviews and Meta-Analyses (PRISMA) guidelines (7). We searched the electronic databases PubMed/MEDLINE, Embase, Cochrane Library, and Web of Science through September 28, 2022. A combination of controlled vocabulary and text words was used. Terms included: “diffusion tensor imaging,” “DTI,” “traumatic brain injury,” “TBI,” and “concussion.” The initial search comprised all TBIs including mild, moderate, and severe. Data was then extracted and curated for this review to focus on mild TBI and our companion paper “The Second Decade of DTI in TBI Part 2: a Systematic Review of Moderate and Severe TBI” to focus on moderate and severe TBI. This process is described in more detail in the sections below. The searches were conducted without any geographical restrictions and were limited to English-language articles. Only articles published between 2012 and 2022 were included since our goal was to provide an updated overview of DTI in TBI studies in the decade since the previous review.

### Study selection

All references were imported into Endnote 20 reference management software (Clarivate, Philadelphia, PA, USA) and de-duplication was carried out. They were then uploaded to Covidence (Veritas Health Innovation, Melbourne, Australia), an online literature review management tool. Further de-duplication was performed, followed by screening of the articles against the eligibility criteria, first based on the title and abstract and then based on the full text. Each article was independently assessed by two reviewers who were blinded to each other's decisions. Conflicts were resolved by the lead reviewers (MFC and FR). Details of the article screening and key decisions were preserved in Covidence. Studies were included in the systematic review if they met the following criteria: (1) peer-reviewed original research; (2) written in English; (3) participants were adults and/or children with TBI of any severity from sub-concussive through severe; and (4) DTI or advanced diffusion imaging [e.g., high angular resolution diffusion imaging (HARDI)] was performed at one or more time points. Exclusion criteria included: (1) articles in languages other than English; (2) studies conducted on animals or *in vitro*; (3) primary disease focus other than TBI (including post-traumatic stress disorder (PTSD), post-traumatic headache and tumors); (4) studies not employing DTI or advanced diffusion imaging; and (5) references that were not research studies (e.g., reviews, editorials, etc.) or that lacked full peer-review (e.g., conference abstracts, protocols, etc.).

### Data extraction and quality assessment

References that passed the screening process underwent data extraction and quality assessment by two members of the review team using a customized form created in Covidence. The data extraction form collected information on the study and participant characteristics—such as study design, setting, participant demographics, injury severity, mechanism of TBI, and imaging details—along with the major outcomes. In addition, a quality assessment form drawing on selected questions from the quality assessment tools developed by the National Heart, Lung, and Blood Institute was created in Covidence and used to evaluate each study.

## Results

**Figure 1:**
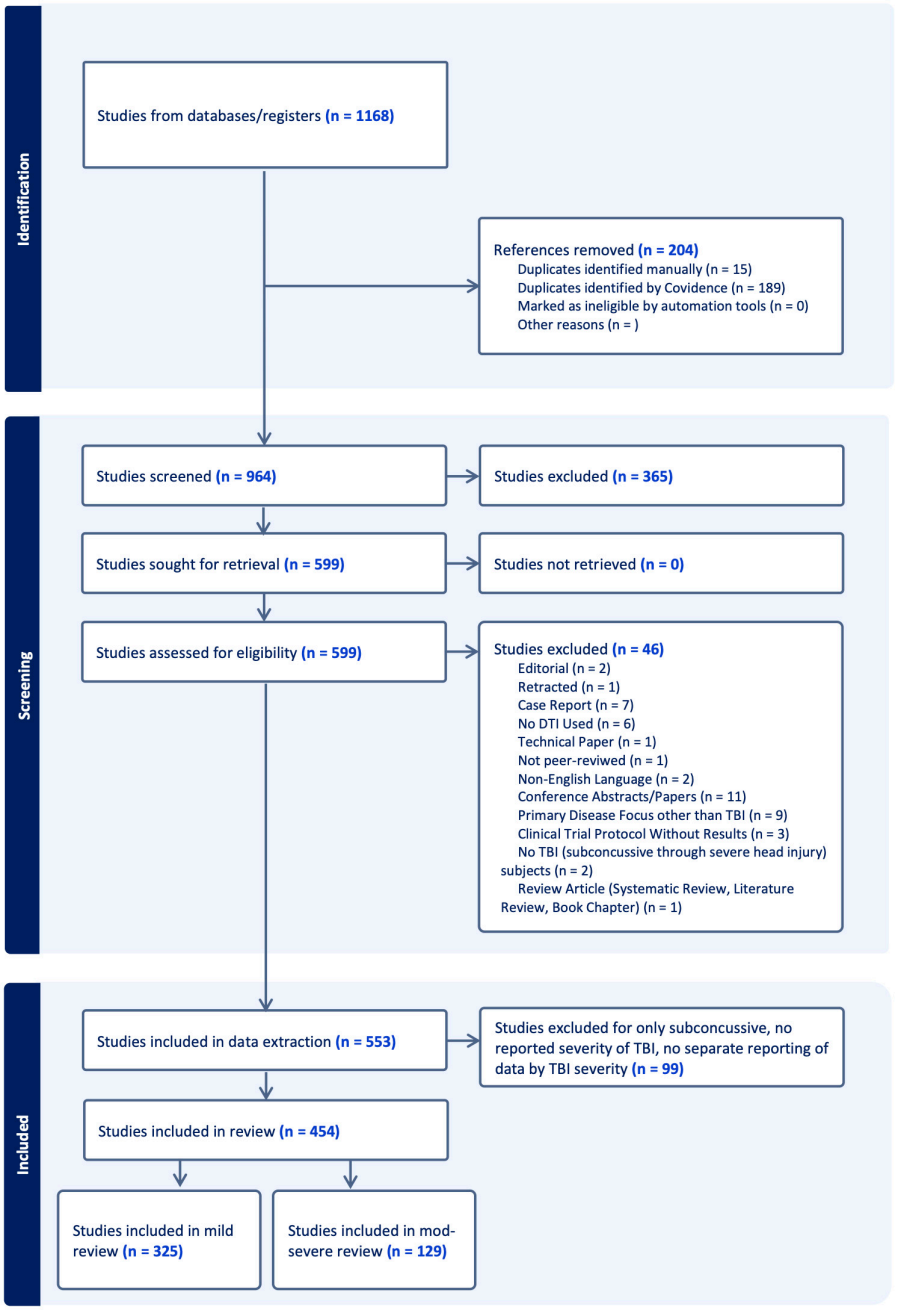
Preferred Reporting Items for Systematic reviews and Meta-Analyses (PRISMA) flow diagram. Results of the initial search, title/abstract screening, and full text review, including reasons for exclusion are presented in the flow chart.

A total of 1,168 articles were imported into Covidence. After removal of 204 duplicates, we screened the title and abstract of 964 studies and excluded 365 of them because they did not meet our inclusion criteria. Full text was reviewed for the remaining 599 studies. Ultimately, 553 studies underwent data extraction and quality assessment. The PRISMA flow diagram is displayed in Figure 1. Among the extracted studies, 330 focused on mild TBI, 34 moderate or severe TBI, and 178 included a range of TBI severity. Eleven studies did not report TBI severity.

The 553 articles included in the extraction phase of the systematic review were further filtered to exclude any studies that only reported on sub-concussive head impacts or did not report any TBI severity of study groups (*n* = 99). The remaining articles were divided into two subgroups: mTBI only (*n* = 325) and Moderate or Severe TBI (*n* = 129). The two subgroups are reported in separate companion papers, with the present paper focused on 325 studies reporting on mTBI. When a study included multiple severities of TBI, but reported results for mTBI separately from msTBI, the study is included among the 325 mTBI studies reported in this review. msTBI findings from the same study are reported in the companion paper on msTBI.

### Publication frequency and study location

**Figure 2:**
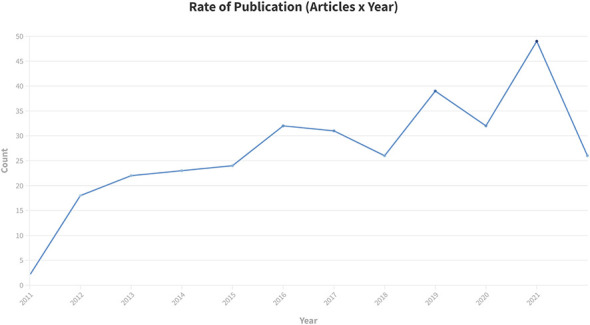
Rate of publication of mTBI articles 2012–2022. The number of studies published per year in this systematic review. This demonstrates and overall increase in the number of published studies of DTI in mTBI year to year over the 10-year inclusion period.

**Figure 3:**
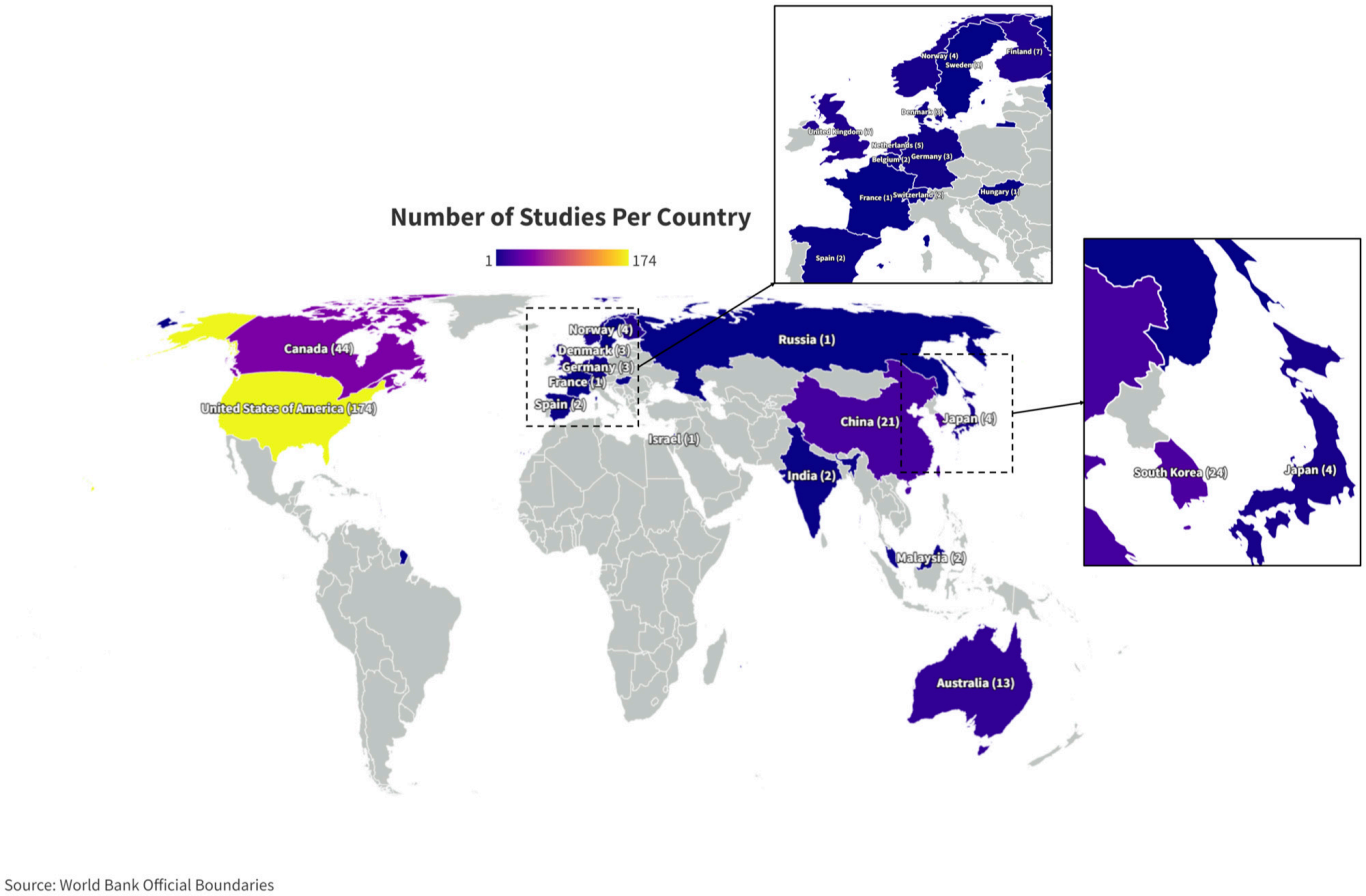
Geographic distribution of mild TBI studies. Country of origin, determined by where each study included in this systematic review took place, is denoted on the world map. The color of the country denotes how many papers took place in that country. The number of studies is included in parentheses. The fewest studies took place in countries colored dark blue, while the most numerous studies took place in countries colored lighter purple and yellow. Parts of the world that are colored gray, without a country name identifier or number in parentheses, did not conduct a study of DTI in mTBI that was included in this systematic review.

Over the past decade there has been an overall increase in the yearly publication rate for mTBI papers (Figure 2). A majority of studies reporting mild TBI were conducted in the United States (Figure 3). No studies included in this review took place in Africa and only a single study took place in either South America or the Middle East. We limited our search results to English language, which may have influenced our ability to include studies from non-English speaking countries. In addition, advanced MRI techniques may not be available in many regions. Multinational research can contribute to health equity as we identify structural and social barriers to mTBI care. Compared to msTBI studies, there is less geographical variability, which may be due to less perceived importance of studying mTBI outside of the US. Identifying individuals for study participation may also be more difficulty if people do not seek care for mTBI.

### TBI patient demographics

An overview of demographic data across the mTBI studies is detailed in Table 1. The 325 studies include 26,287 mTBI participants, compared to 9,609 msTBI participants across 129 studies. More men (70.50%) than women were included as participants in mTBI studies, which reflects more gender parity compared to msTBI studies, where 76% of participants were men. Approximately 26% of 325 mTBI studies specifically recruited athletes, whereas no sport populations were specially recruited in studies of msTBI in 2012–2022. We cannot exclude the possibility that some patients may have been included in more than one sample, as in the case of studies published by the same authors which reported similar patient sample characteristics. In addition, 61 of 325 (18.77%) studies included participants under the age of 18, for a total of 3,153 participants. On average, 30.47% of participants under the age of 18 were female. The outsized proportion of men included in mTBI studies is likely due to recruitment of athlete participants in male predominant sports (i.e., football) and military service members. In addition, epidemiologic studies have demonstrated that more men sustain mTBI. This may underestimate the amount of woman sustaining mTBI as the result of intimate partner violence, which affects one in three women in their lifetime (8). Up to 75% of woman experiencing intimate partner violence will experience TBI. In addition, emerging evidence demonstrates that woman may be more susceptible to adverse outcomes following TBI (9). Therefore, it is important to prioritize recruitment of women in studies of mTBI. Variable inclusion of demographic features and mechanism of injury in individual studies limits integration of patient data and inferences targeted to specific features or mechanisms.

**Table 1:** Overview of demographic data for included mTBI studies.

Demographic variables	Value
Study subjects	
Total mTBI participants	26,287
Average mTBI participants per study	81
Range of mTBI participants per study	2–367
Sex	
Male	70.50%
Female	29.50%
Age	
Age range (years)	9.5–66.8
Average age (years)	30.8
Number of studies with subjects < 18 years old	61
Population studied	
General/Civilian	64%
Sports	26.20%
Military	12%
Unspecified	1.20%
Mechanism of injury	
MVA, falls, assaults	25.23%
Sports	23.69%
Military blasts	6.77%
Mixed: MVA, falls, assaults, Sports, Blasts	23.08%
Not reported	21.23%

Number of patients, sex, age, population, and injury mechanism are described in the table. Blasts refer to mTBI sustained due to explosions in the military setting. Mixed mechanism of injury includes studies with patients who sustained mTBI due to a combination of motor vehicle accidents (MVA), falls, assaults, sports, or blasts.

### Severity, chronicity, and study design

Severity of TBI was most commonly determined by Glasgow Coma Scale (GCS; mild: GCS 13–15, moderate: GCS 9–12, severe: GCS 3–8). However, various clinical mTBI definitions were used to determine whether participants sustained mTBI. These include VA/DoD practice guidelines (10), World Health Organization's Collaborating Center for Neurotrauma Task Force definition (11), American Congress of Rehabilitation Medicine definition (12), and Concussion in Sport Consensus guidelines (13). These guidelines have largely converged to define mTBI as an impact to the head resulting in neurologic symptoms including alteration of consciousness, with loss of consciousness not exceeding 30 min, post-traumatic amnesia not exceeding 24 h and/or GCS 13–15. We assumed the reported severity assignment was accurate. However, bias of participant inclusion due to the definition used cannot be excluded.

Similar to early studies of TBI with DTI, there were several articles that distinguished between mild (GCS > 13, absence of imaging findings) and mild-complicated (GCS > 13, presence of imaging findings) TBI (6). However, many articles categorized mild-complicated patients with moderate-severe patients, as the clinical features may be more closely related to this group compared to the mild-uncomplicated TBI group. If mild-complicated TBI patients were categorized as moderate-severe TBI, they would not be included in this review of mTBI. Papers reporting on mTBI, which included complicated mTBI patients in their cohorts would be included in this review. Seven out of 325 studies (2.15%) explicitly compared complicated mTBI to uncomplicated mTBI (14–20). Four of seven studies found no difference in DTI measures between uncomplicated and complicated mTBI (17–20), while three studies reported more extensive white matter changes complicated mTBI (14–16).

**Figure 4:**
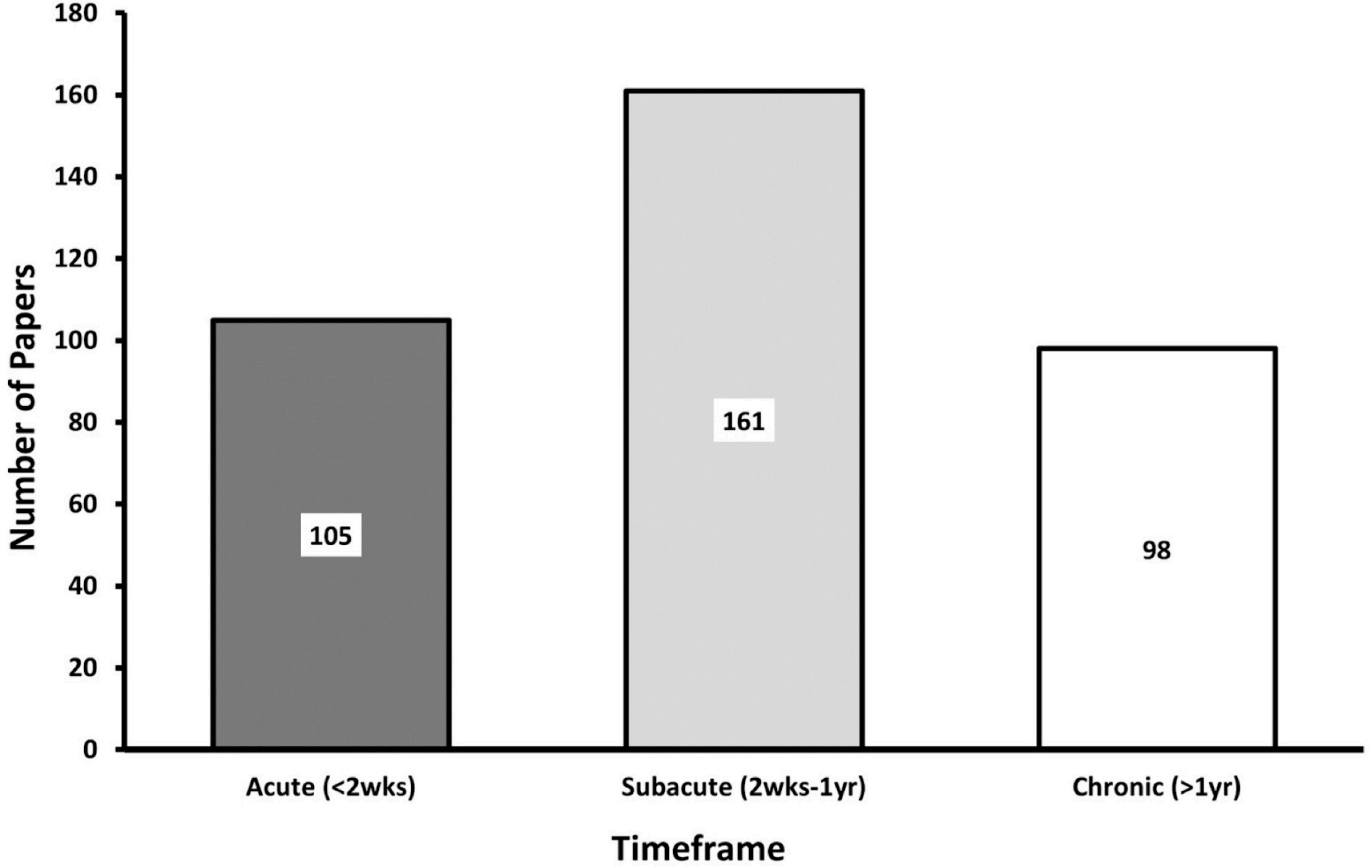
Post-injury DTI acquisition. This bar graph denotes when DTI was acquired in the included studies. Studies were only included if there was sufficient information to determine the chronicity of individual patient injuries. Studies may be included in more than one category if they studied patients at multiple timepoints. Thus, the total number of studies represented in this graph exceeds the total of included papers (Acute: 0–2 weeks, Subacute: 2 weeks−1 year, Chronic: >1 year).

Timing of study assessments after mTBI varied across papers. We classified papers according to three periods following TBI: acute (< 2 weeks), subacute (2 weeks−1 year), and chronic (>1 year). Most mTBI papers reported on the subacute phase of injury, similar to 2002–2012 (Figure 4) (6). The popularity of the subacute time point may be logistically motivated as identification, screening, and enrollment within the brief acute period is challenging. By contrast, msTBI studies most often occurred in the chronic phase of injury. Microstructural pathology evolves over time following injury. Therefore, the point in time after injury during which DTI is obtained is crucial for appropriate comparisons.

**Figure 5:**
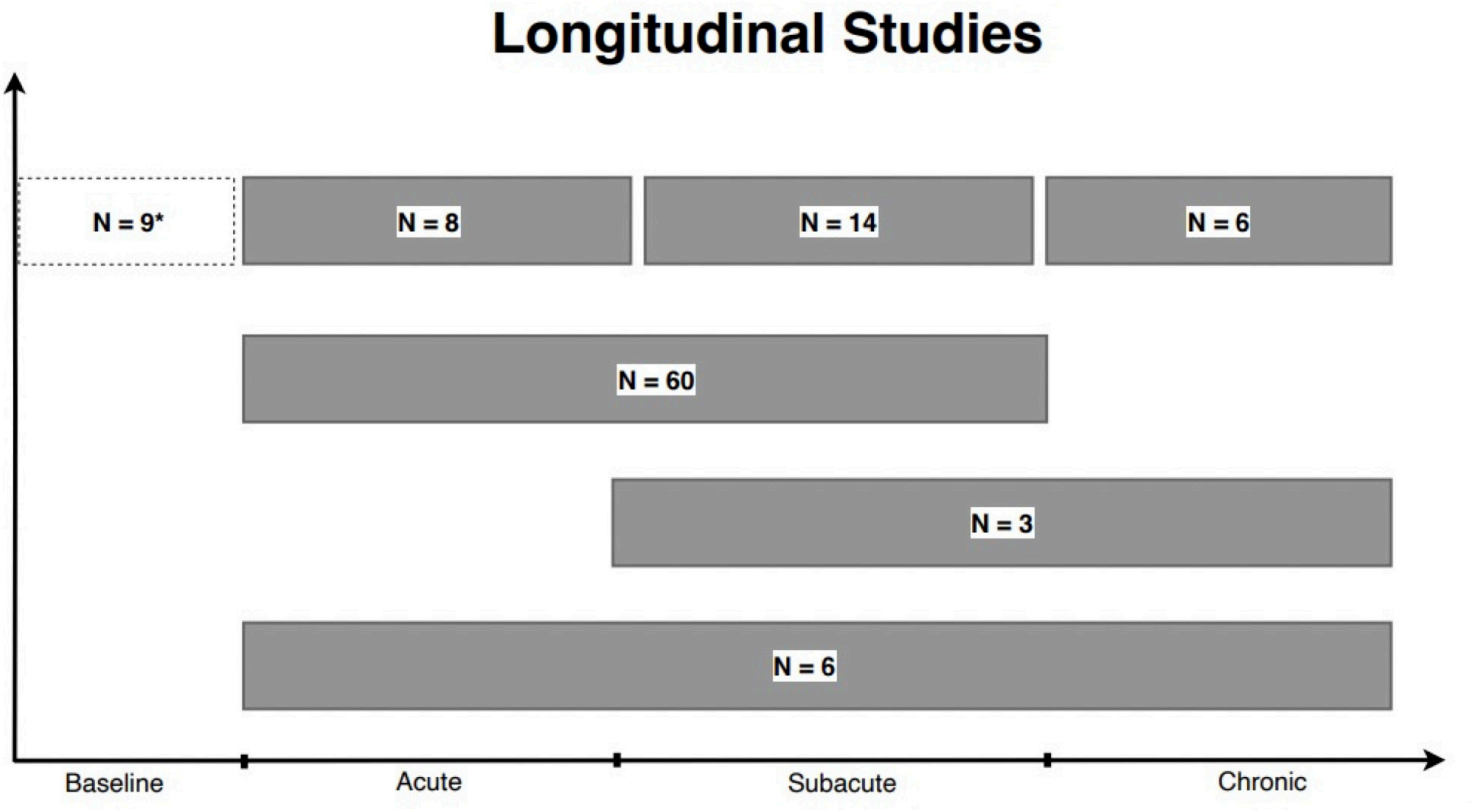
Longitudinal studies. A total of 97 studies reported longitudinal data. “*N*=” represent the number of studies within each grouping. Six studies examined patients in the acute, subacute, and chronic phases of TBI. Three studies examined patients in the subacute to chronic phase while sixty studies examined patients within the acute to subacute phase. Finally, 8, 14, and 6 studies obtained a DTI scan on the same patient more than once in the acute, subacute, and chronic phases, respectively. *Baseline represents the number of studies including a scan prior to injury among longitudinal studies. This number is not added to the total of longitudinal studies.

29.85% of the 325 studies evaluated patients at multiple time points throughout the course of the study (Figure 5) (14, 15, 21–115). While longitudinal studies are often difficult to conduct due to cost and participant drop-out, they provide important information on potential recovery. 29.85% of studies is a substantial increase in longitudinal studies compared to the 2002–2011 decade, where 13% of mTBI and msTBI studies including DTI acquisition at multiple time points (6).

6.15% of the 325 studies utilized a case-control study design (39, 96, 116–133), 4.92% of studies utilized a cross-sectional study design (71, 134–148), 1.54% of studies utilized a before-after study design (52, 77, 100, 102, 109), 1.23% used a randomized controlled study design (37, 53, 54, 69), with the remaining publications using a cohort study design. More diverse study designs were included in the current decade compared to 2002–2011. However, for both msTBI and mTBI >80% of studies used a cohort study design.

11.08% of 325 mTBI studies did not use a control comparison group (20, 27, 33, 37, 52, 53, 63, 65, 70, 77, 86, 100, 102, 107, 109, 140, 143, 144, 147–164). Of studies that used a control group, 40.48% compared participants with mTBI to healthy controls, 9.69% compared to military controls, 10% compared to orthopedic injury controls, and 14.53% compared to athletes without concussion. Aside from these major categories, two articles compared participants with mTBI to participants who had sustained emotional trauma or presented with neurologic complaints (headache) without history of TBI (165, 166). Articles that did not use a separate control group, either tested associations of DTI metrics with clinical or cognitive outcomes or compared the same participant's DTI metrics before and after a season of sport engagement. Studies also frequently included subgroup analyses to further explore comorbidities of mTBI. For example, mTBI vs. mTBI with post-concussion syndrome (PCS), mTBI vs. mTBI with post-traumatic stress disorder (PTSD), mTBI vs. mTBI and depression, and uncomplicated mTBI vs. complicated mTBI. When evaluating studies, the control group is an important feature. Ideally, the control group is as close demographically and physically to the mTBI group, and therefore using military and athlete controls accounts for physical activity and similar lived experiences compared to the head injured group.

### Data acquisition parameters

269 (82.8%) of the 325 studies utilized a 3T MRI scanners (14–56, 58, 59, 61, 62, 64, 66–82, 84–96, 98–109, 111–125, 128, 130–134, 137–150, 152–158, 160, 161, 165–299), 47 (14.5%) used 1.5 T (57, 110, 126, 127, 129, 135, 136, 151, 159, 162–164, 300–333), 1 (0.31%) used 7T (334), and 6 (1.85%) did not report magnetic field strength (60, 63, 65, 83, 335, 336). There was a large increase in the use of 3T magnets for DTI research in the current decade compared to the previous decade, in which 3T and 1.5T magnets were used in equal numbers (6). This is likely due to the increasing availability and affordability of high strength magnets. Greater magnetic field strength provides enhanced signal to noise ratio (SNR), which can be leveraged to shorten acquisition time while enhancing spatial resolution and/or increasing the number of diffusion-sensitizing directions, potentially allowing smaller, more subtle microstructural alterations to be detected.

Eighteen studies did not report the *b*-value employed (19, 38, 40, 60, 63, 65, 83, 91, 93, 97, 109, 196, 203, 261, 287, 301, 308, 323). Of the 307 studies that reported *b*-values, 266 (81.8%) out of 307 were single-shell studies (using one unique non-zero *b*-value), with a *b*-value ranging from 600 to 3,000 s/mm^2^ (14–18, 20–28, 32– 35, 37, 41, 43–45, 47, 49–51, 53–59, 61, 62, 64, 66, 68–70, 73–76, 78, 79, 84–90, 94–96, 98–103, 105– 107, 110–123, 126–130, 133–136, 138–148, 150–159, 161, 165–178, 181, 183–192, 195, 197–202, 204, 206, 208–212, 214–218, 220–229, 231–237, 240–242, 245, 246, 248, 250–252, 254–260, 263–276, 278– 285, 288–293, 300, 302–307, 309–322, 324–332, 335–338) (80–82, 131, 132, 162–164, 262, 295–299, 333). Of the 307 studies that reported *b*-values, there were 41 (12.6%) muti-shell studies (using several unique non-zero *b*-values), with *b*-values ranging from 100 to 6,250 s/mm^2^. Of the 41 multi-shell studies, 31 studies utilized two *b*-values (29–31, 36, 39, 42, 46, 48, 52, 67, 71, 77, 92, 104, 108, 124, 125, 160, 179, 180, 182, 193, 194, 205, 207, 213, 219, 238, 247, 277), three studies utilized three *b*-values (72, 230, 286), two studies utilized four *b*-values (149, 334), and five studies utilized five *b*-values (137, 243, 244, 249, 294). A subset of articles used multi-shell diffusion techniques, such as Neurite Orientation Dispersion and Density Imaging (NODDI), which require multiple *b*-values. The *b*-value is a parameter that reflects the strength and timing of the diffusion-sensitizing gradient magnetic fields, with higher *b*-values resulting in greater diffusion-related signal effects, but lower SNR (339). While the majority of studies used a single *b*-value, multi-shell techniques are being more used more frequently, as evidenced by the increasing number of studies employing advanced diffusion methodologies compared to the previous decade (6).

The reported number of diffusion-sensitizing directions across 309 studies ranged from 6 to 200, with a mean of 42. 16/325 studies did not report the number of directions (38, 60, 72, 83, 91, 124–126, 182, 196, 261, 287, 305, 315, 319, 335). Increasing the number of diffusion-sensitizing directions can increase the accuracy of diffusion scalar and diffusion direction estimates, but at the cost of additional image acquisition time (339, 340). The average number of diffusion-sensitizing directions is higher compared to the previous decade, during which the average value was 27 (6).

**Figure 6:**
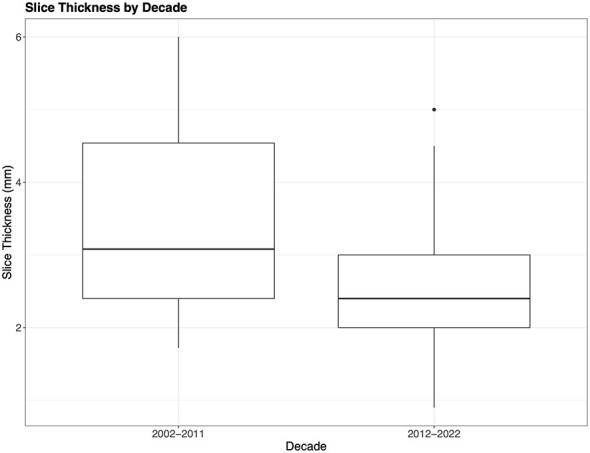
Slice thickness by decade. The boxplots show the difference in acquisition slice thickness from the initial decade compared to the most recent decade. Notably Decade 1 (2002–2011) includes mild-severe TBI (3.08 mm), while Decade 2 (2012–2022) only indicates slice thickness for mild TBI (2.54 mm).

With respect to slice thickness, the mean reported value was 2.54 mm (range 0.9–5 mm) among 241 articles that reported the slice thickness. Eighty-four articles did not report this information (14, 16, 19, 21, 22, 29–33, 35, 37, 38, 46, 47, 55, 60, 65, 67, 70, 72, 82, 83, 86, 91, 93, 95, 97, 100–102, 107–109, 121, 127, 132, 137, 140–142, 147, 149, 154, 155, 158, 165, 169, 170, 175, 179, 184, 185, 189, 196, 210–213, 221, 222, 232, 235, 237, 248, 250, 261, 264, 266, 267, 273, 275, 276, 281, 286, 294, 296, 301, 302, 308, 317, 321, 323, 334). As the slice thickness decreases, the axial resolution of the images increases and the SNR decreases (339). Studies during the past decade have used, on average, thinner slice thickness (2.54 mm compared to 3.08 mm) compared to the previous decade affording investigators better spatial resolution (Figure 6) (6).

When evaluating the use of DTI across several studies, it is important to consider the different imaging parameters utilized including strength of the magnetic field, number of diffusion-sensitizing directions, choice of *b*-values, and choice of slice thickness. The ability to consider these factors starts with clear reporting of imaging parameters. Understanding the impact of imaging parameters is essential to ensure the validity, reliability, and interpretability of results, as well as for advancing the field toward standardized and optimized imaging protocols. Despite differences in acquisition parameters, data harmonization across sites and studies is possible. Initiatives like ENIGMA have created protocols to allow for harmonization across sites and scanners to create large datasets with greater power for detecting differences and for use in genomics studies (341, 342).

### Data analysis methods

During the past decade, studies primarily used either a region of interest (ROI) or whole-brain approaches. The ROI method entail *a priori* specification of a region or WM tract of interest, either manually delineated or derived from a canonical atlas, from which diffusion scalar measures are extracted for further analysis (339). A ROI approach allows for hypothesis-driven testing of specific brain regions (339). This approach is appropriate for testing associations of specific WM regions with cognitive or behavioral measures, to address specific functional- or injury mechanism-related hypotheses. An important limitation of region-focused analyses is that TBI is spatially heterogeneous depending on each participant's injury. While some brain structures, such as the corpus callosum, have been reported as more prone to injury, these results are subject to selection bias inherent in pre-specified ROI. Whole-brain approaches, such as voxel-wise analyses do not impose an *a priori* regional restriction, allowing detection of abnormality throughout, for example, the brain white matter (339). Voxel-based approaches are also automated and provide greater spatial resolution, which can enhance sensitivity to smaller abnormalities, compared to the use of larger *a priori* ROI (339). Although whole brain analyses avoid the potential limitation of pre-specified ROI, group comparisons, even when conducted with a whole brain analytic approach, are still subject to bias, in that only regions that are similarly affected by TBI will be detected as abnormal. Since TBI is highly spatially heterogeneous, it is likely that individuals will not all exhibit pathology in the same location. Injury that occurs at a given location in few or individual patients, will not be detected in the group comparison. To avoid this issue, individualized whole brain analyses have been developed, which compare each individual patient to a control group to delineate where that individual exhibits microstructural injury.

In our systematic review of 325 mild TBI articles, 192 studies used ROI analysis. Among those 192 studies, 42 studies using manual methods (18, 37, 39, 47, 60, 71, 72, 89, 103, 113, 114, 117, 122, 133, 139, 142, 157, 160, 162, 169, 175, 179, 196, 201, 202, 217, 227, 246, 248, 254, 257, 281, 285, 286, 292, 298, 301, 309, 319, 323, 337, 343) and 150 studies used atlas-derived methods (14–17, 19–21, 23–26, 28, 29, 32–34, 36, 40, 42–45, 48, 49, 51, 53–56, 58, 62, 63, 69, 74, 75, 84, 85, 87, 91, 96, 101, 102, 104, 105, 108, 110, 112, 118, 120, 121, 123, 124, 126, 130, 131, 134–136, 138, 141, 148–150, 152, 153, 155, 156, 158, 161, 163, 164, 167, 168, 170, 172, 174, 178, 180, 188, 189, 191, 192, 197, 198, 203– 205, 209, 210, 213, 218, 222, 225, 226, 229, 231, 232, 234, 235, 240, 243, 247, 250, 251, 253, 255, 256, 258, 259, 261, 263, 265, 268, 271, 274, 277, 280, 282, 283, 289, 294–297, 303–308, 311–315, 317, 318, 324–335, 338). For the 42 manually drawn studies, only 21/42 (50%) reported reliability testing (39, 71, 89, 113, 114, 122, 133, 139, 142, 175, 179, 201, 202, 217, 227, 246, 254, 285, 292, 301, 337). Two hundred and two of the 325 studies used whole-brain analysis with 200 of the 325 studies using voxelwise/TBSS approach (14, 16, 18–20, 22, 25, 27–32, 35, 36, 38, 40, 42, 43, 45–48, 50–52, 55–61, 64–66, 70–73, 75– 84, 86, 88–95, 98–100, 106–109, 111, 112, 115, 116, 119, 120, 125–132, 137, 140, 143–147, 150–152, 154, 155, 163–167, 169, 171, 173, 176, 177, 180–187, 189, 190, 192–195, 197, 199–201, 204, 206–210, 212–216, 219, 220, 223–225, 228, 230, 231, 233, 234, 236–238, 240–245, 249, 251–254, 256–258, 260, 262–270, 272– 279, 282, 284, 285, 287, 288, 290–294, 296, 299, 300, 302, 304, 310, 316, 320–322, 331, 332, 336, 338), and two using histogram analysis (280, 289). Fifteen studies used other proprietary or individually developed analysis methods (34, 41, 44, 67–69, 97, 153, 159, 188, 211, 221, 233, 237, 324). Seventy-six studies used a combination of ROI and whole-brain analysis (14, 16, 18–20, 25, 28, 29, 32, 36, 40, 42, 43, 45, 47, 48, 51, 55, 56, 58, 60, 71, 72, 75, 84, 89, 91, 108, 112, 120, 126, 130, 131, 150, 152, 155, 163, 164, 167, 169, 180, 189, 192, 197, 201, 204, 209, 210, 213, 225, 234, 240, 243, 251, 253, 254, 256–258, 263, 265, 268, 274, 277, 280, 282, 285, 289, 292, 294, 296, 304, 331, 332, 338). Single subject analysis was used in 9.54% of the 325 studies (32, 33, 40, 50, 52, 58, 61, 67, 70, 77, 102, 104, 115, 126, 131, 135, 148, 150, 159, 183, 217, 235, 240, 246, 272, 273, 283, 317, 333, 337, 338).

The past decade has seen an increasing proportion of studies reporting whole brain analysis methods. This pattern may reflect a growing appreciation for the spatial and inter-individual heterogeneity of TBI pathology, reflecting both injury and individual characteristics, and the potential limitations of specifying ROI based upon *a priori* hypotheses.

### Specific diffusion measures studied

DTI models the diffusion-weighted MRI signal from each image voxel to generate quantitative metrics, including measurement of the degree of anisotropy and dominant direction of diffusion (339). In the DTI model, the diffusion process is modeled as an ellipsoid defined by three vectors (λ1, λ2, λ3). These three vectors can be used to generate multiple quantitative measurements at each voxel, which include but are not limited to fractional anisotropy (FA, directional coherence of water), mean diffusivity/apparent diffusion coefficient (MD/ADC, total direction-independent diffusion), radial diffusivity (RD, a measure of average diffusion along the two minor axes of the diffusion ellipsoid), axial diffusivity (AD, a measure of diffusion along the principal axis of the diffusion ellipsoid). These measures indirectly reflect the microstructural organization of brain white matter and can be used to assess microstructural changes and integrity of tissue.

FA was by far the most commonly studied DTI scalar measurement across all of the reviewed articles, reported in 312 out of 325 articles (96%) (14–34, 36–40, 42–52, 55–67, 69–85, 87–89, 91–106, 108–148, 150–167, 169–174, 176–190, 192–238, 240–289, 291–318, 320–338). MD was the second most common DTI scalar measurement studied (180/325 or 55.38%) (14–21, 23–27, 29, 30, 32, 34, 36–39, 42, 44–48, 50, 55, 58–62, 64–67, 70, 71, 73, 77, 78, 80–82, 84, 88, 92–95, 97, 99, 101, 102, 104–109, 111–114, 119–123, 128, 130, 131, 133, 137–139, 142, 145, 148, 150–154, 157, 158, 160, 161, 163, 164, 166, 169, 170, 173, 176, 180–183, 185, 186, 189, 192, 193, 195, 197, 198, 201, 202, 205, 206, 209–215, 218, 219, 221, 222, 225–227, 230, 232–234, 236, 238, 240, 242–250, 252, 253, 259–261, 263, 265, 266, 272, 274, 276–279, 281–284, 287, 288, 291, 293–297, 299, 301, 313, 316, 317, 321–324, 334, 338), and less commonly studied were RD (140/325 or 43.08%) (14–20, 27–29, 31, 33, 37–39, 42–44, 46–48, 51, 55, 56, 58–62, 64, 65, 67, 70, 73, 76, 82, 84, 86, 93–97, 100–102, 105, 108, 109, 111, 112, 114, 119, 120, 126–128, 130, 131, 134, 137–139, 145, 151, 154, 157, 160, 162, 164–166, 170, 173, 179–181, 183–186, 188, 189, 193, 195, 197–199, 205, 206, 209–211, 213, 216, 218–220, 223, 227, 230, 232, 234, 236, 241, 243, 244, 246, 248, 252, 253, 256, 258, 260, 261, 263, 265, 266, 268, 276–279, 282, 284, 287, 291, 292, 294–296, 298, 299, 301, 302, 316, 317, 322, 336, 338), AD (133/325 or 40.09%) (14–20, 27–29, 31, 33, 37, 38, 42–44, 46–48, 51, 55, 56, 59–62, 64–66, 70, 73, 76, 82, 84, 86, 94–97, 100–102, 105, 108, 109, 111, 112, 114, 119, 120, 126–128, 130, 131, 134, 137–139, 145, 151, 154, 157, 160, 162, 164–166, 170, 173, 179–181, 183–186, 188, 189, 193, 195, 197–199, 205, 206, 209–211, 213, 216, 218–220, 223, 227, 232, 234, 236, 241, 243, 244, 248, 252, 253, 256, 258, 260, 261, 263, 265, 266, 276–278, 282, 284, 287, 291, 292, 294–296, 298, 299, 301, 302, 316, 317, 336, 338), and ADC (29/325 or 8.92%) (33, 56, 72, 85, 117, 135, 136, 162, 174, 175, 179, 184, 199, 203, 217, 223, 286, 298, 303, 306, 312, 315, 318, 319, 325, 326, 335–337). 20.7% of the 325 studies reported additional measures from advanced diffusion methods, such diffusion-based connectivity (6.77%) (35, 49, 54, 57, 66, 68, 69, 79, 84, 90, 110, 141, 149, 150, 168, 178, 191, 196, 221, 229, 276, 290), diffusion kurtosis imaging (DKI; 7.69%) (21, 29, 37, 39, 42, 46, 48, 52, 71, 108, 137, 142, 160, 182, 202, 207, 213, 219, 238, 249, 277, 283, 294, 334, 344), and NODDI (2.15%) (31, 92, 179, 205, 212, 244, 277).

**Figure 7:**
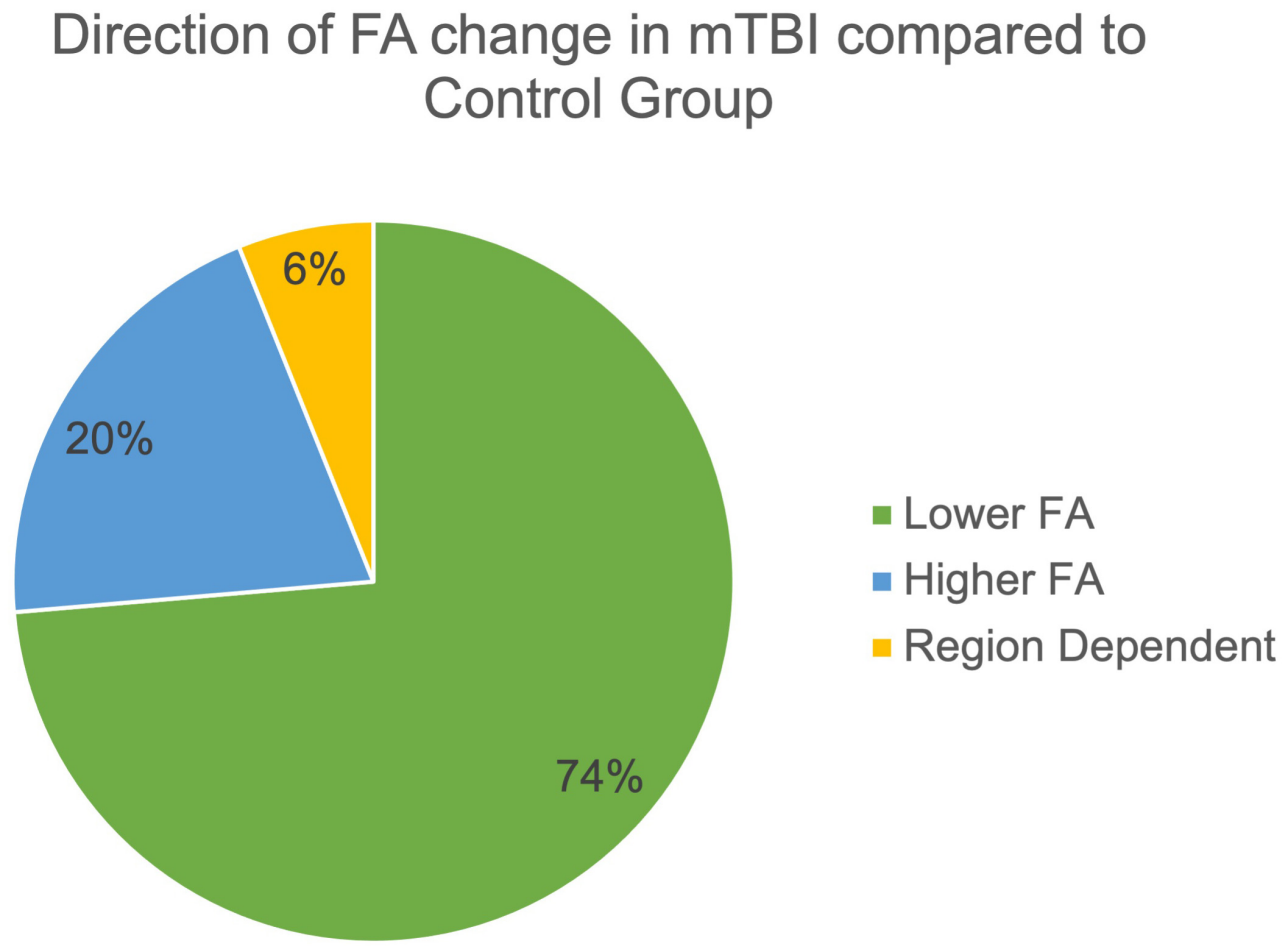
Significant group differences in FA. The pie chart demonstrates of the 148 studies that found significant group wise differences in FA between mTBI participants and controls, that 109 found lower FA in the mTBI group, 30 found higher FA in the mTBI group, and nine found regional variation in FA.

Of the 289 studies that compared DTI scalar metrics in mTBI with controls, 219 (75.78%) reported significant group-wise differences in one or multiple diffusion derived metrics (14–17, 19, 21, 22, 24–32, 34, 36, 38, 42–47, 51, 54–58, 60, 64, 68, 69, 71–73, 75, 76, 78, 79, 81–83, 85, 88, 89, 91–95, 98, 99, 101, 104–106, 111–113, 115, 117, 119, 120, 122–129, 131, 133, 135, 136, 138, 141, 145, 146, 150, 163–167, 170, 173, 175, 178–181, 183–190, 192, 194–196, 198, 200–204, 207–220, 224–228, 230–233, 235, 236, 238, 240–244, 246, 248–254, 256, 257, 259–261, 263, 265–273, 276–282, 285–292, 294–296, 298–302, 304, 309–312, 314–322, 324, 326–334, 336–338). There were 148 studies that demonstrated statistically significant group differences in FA. Of those, 109 (73.6%) out of 148 found that FA was lower in mTBI groups, 30 (20.3%) found FA was higher in mTBI groups, and nine (6.1%) found that FA was significantly higher or lower in mTBI group depending on the time point or brain region assessed (Figure 7). Overall, these findings are consistent with findings in msTBI compared to controls, particularly that those with head injury are found to have lower FA, but there is clearly more variability among the findings of mTBI studies. In studies that demonstrated significant group differences in FA among msTBI patients compared to controls, 98.7% reported lower FA in the msTBI group, compared to 73.6% in mTBI. Injury severity, brain regions investigated, and time post-injury likely have a significant effect on the direction of the FA abnormality. In some studies included, there are regions of the brain where FA is found to be higher compared to controls while there are other regions of the brain where FA is found to be lower compared to controls. Elevation of FA is thought to represent edema, where axonal swelling compresses the extracellular space resulting in more diffusion restriction, while low FA is thought to represent axonal and myelin loss resulting in less restricted diffusion due to loss of tissue constituents. It has also been proposed that high FA could represent a compensatory reaction to injury, which may be more likely to occur in mTBI compared to msTBI (61). In mTBI in pediatric populations, low FA is more likely to represent temporarily disrupted ongoing development processes as opposed non-reversible loss of axons or myelin, as these changes in longitudinal studies have been dynamic (22).

Of 73 studies that reported significant group differences in MD or ADC, 54 (74%) of 73 found significantly higher MD/ADC, while 19 (26%) found significantly lower MD/ADC in those with mTBI. MD and ADC typically exhibit opposite directionality in comparison to FA. The proportions of studies reporting higher MD/ADC is similar to the proportion reporting lower FA. As with FA, MD/ADC findings in mTBI were more variable than in studies of msTBI, where 92.3% of studies reporting on MD, found higher MD in msTBI patients.

The brain region most commonly found to exhibit a significant difference of DTI measures compared to controls was the corpus callosum (17, 21, 28, 32, 47, 56, 60, 72, 75, 78, 85, 104, 105, 113, 117, 124, 133, 138, 167, 175, 186, 189, 192, 202, 217, 218, 225, 226, 232, 240, 246, 253, 257, 259, 286, 290–292, 294, 295, 298, 301, 312, 337, 338, 344). In addition, several studies reported significant results in the longitudinal fasciculus (14, 17, 19, 24, 28, 60, 101, 133, 138, 167, 189, 192, 213, 218, 226, 230, 232, 240, 257, 272, 294, 304, 333, 338), internal capsule (19, 28, 32, 75, 101, 189, 192, 202, 217, 253, 259, 292, 294, 298, 301, 304, 333, 337, 338, 344), external capsule (19, 32, 167, 189, 202, 304, 333, 338), corona radiata (19, 28, 43, 72, 78, 101, 105, 123, 138, 167, 189, 192, 202, 217, 259, 286, 294, 304, 333, 337, 338), thalamic radiation (14, 19, 24, 123, 138, 189, 213, 218, 231, 232, 291, 304), thalamus (21, 45, 51, 69, 71, 72, 104, 202, 282, 286, 318, 344), cingulum (14, 15, 17, 19, 24, 43, 56, 60, 85, 101, 113, 124, 138, 167, 189, 192, 213, 218, 232, 272, 291, 292, 294, 301, 304, 314, 333, 338), inferior fronto-occipital fasciculus (19, 24, 101, 138, 167, 192, 213, 218, 224, 230, 240, 259, 272, 333, 338), uncinate fasciculus (14, 15, 17, 19, 24, 28, 47, 60, 85, 101, 113, 122, 124, 133, 175, 189, 213, 217, 218, 230, 232, 240, 253, 259, 291, 304, 337), corticospinal tract (14, 19, 24, 47, 189, 213, 218, 232, 240, 295, 304, 314, 329), fornix (14, 17, 19, 60, 101, 124, 189, 203, 226, 292, 304, 314, 330), and cerebral peduncle (19, 43, 133, 167, 189, 217, 301, 304, 333, 337). While the vast majority of these regions of interest are white matter regions, some studies investigated gray matter regions as well. The thalamus was the most common gray matter region with significant differences in DTI measures between those with mTBI and controls. Because of substantial variability in the pre-selected ROIs used across studies, the prevalence of findings within a given region is subject to the frequency at which that region was tested. This represents a potential source of selection bias and an opportunity to standardize future studies in order to enhance combined analyses across studies.

### Advanced diffusion techniques studied

Advanced diffusion imaging techniques, such as diffusional kurtosis imaging (DKI), and neurite orientation dispersion density imaging (NODDI) are not the primary focus of this review. However, use of advanced diffusion techniques has increased in the past decade. 18.77% (61/325) of studies reported results for advanced neuroimaging techniques. Twenty-five of the 61 studies that implemented advanced techniques used DKI, 22 of 61 used diffusion derived connectivity, and seven of 61 used NODDI. All studies that used NODDI also reported DTI measurements.

DKI characterizes non-gaussian diffusion behavior more accurately than DTI (345), and NODDI, characterizes diffusion within the intracellular, extracellular, and free water compartments to provide a more precise biophysical model of tissue water diffusion (346). Diffusion-based connectivity analysis maps neuronal structural connections across brain networks to provide insight into function and disease. These techniques were used in a greater proportion of mTBI studies (18.77%) compared to msTBI studies (11.63%).

### Associations of DTI with patient outcomes

Many studies (228/325, 70.15%) examined relationships of patient outcome measures with DTI measures, most commonly FA and MD (17–83, 124–133, 139–148, 151–166, 201–238, 240–299, 311–334, 336–338). A similar proportion of mTBI studies reported relationships of DTI with clinical outcomes as in the first decade of DTI use in TBI (72%) (6). Patient outcomes explored included cognitive measures, functional outcome scales, symptoms, and other imaging measures. As in the first decade, the two largest categories of outcomes were clinical outcome measures and cognitive function.

Clinical outcome measures included post-concussive symptoms, balance measures, and mood symptoms, as detailed in Table 2. Post-concussive symptoms were quantified with symptom scales, such as Sports Concussion Assessment Tool (SCAT2-5), imPACT, Rivermead Post-Concussion Questionnaire (RPQ), Post-Concussion Symptom Inventory (PCSI), or Post-Concussion Symptom Scale (PCSS). Fifteen of 325 studies of mTBI reported attempting to find associations between FA and post-concussive symptom scales (31, 47, 55, 81, 166, 206, 208, 230, 247, 250, 269, 270, 286, 296, 332). The majority, 9/15 studies, did not find an association between FA and post-concussive symptoms (47, 55, 166, 206, 230, 247, 269, 270, 296). In those that did find a significant association, 4/6 found that lower FA was associated with worse symptoms (31, 208, 286, 332), while 2/6 found that higher FA was associated with worse symptoms (81, 250). Findings for MD were more mixed with three studies finding higher MD was associated with worse symptoms (55, 81, 247), four studies finding lower MD was associated with worse symptoms (29, 46, 250, 296), and two finding no association (47, 206). None of the four studies that attempted associations between FA or MD and balance measures found a significant association. More mood symptoms, including anxiety, depression, and PTSD, measured with symptom checklists (Beck Depression Inventory, Hamilton Rating Scale for Depression, PTSD Checklist, or Post-Traumatic Symptom Scale) were found to be associated with lower FA in three of the seven studies that attempted to find an association (29, 46, 281). The remainder did not find a significant association. While there appears to be a trend toward more concussion and mood symptoms associated with lower FA, these studies are heterogenous, and further large studies are required to further confirm these associations. In comparison to msTBI, where many of the studies focused on global outcomes, patients with mTBI have less physical disability, and the spectrum of clinical outcomes is more focused on symptoms than on physical function.

**Table 2:** DTI associations with clinical outcomes.

DTI measure	Association	Post-concussive symptoms	Balance	Mood symptoms (Depression/PTSD)
FA	Positive	*More symptom ->, higher FA* 2 (81, 250)	*Worse balance -> higher FA*	*More symptoms -> higher FA*
	Negative	*More symptom ->, lower FA* 4 (31, 208, 286, 332)	*Worse balance -> lower FA*	*More symptoms -> lower FA* 3 (206, 269, 285)
	None	9 (47, 55, 166, 206, 230, 247, 269, 270, 296)	2 (230, 281)	4 (153, 166, 208, 274)
MD	Positive	*More symptom ->, higher MD* 3 (55, 81, 247)	*Worse balance -> higher MD*	*More symptom -> higher MD* 1 (206)
	Negative	*More symptoms -> lower MD* 4 (29, 46, 250, 296)	*Worse balance -> lower MD*	*More symptoms -> lower MD*
	None	2 (47, 206)	3 (29, 46, 281)	1 (153)

Studies below attempted to find associations between DTI measures and clinical outcomes. The most commonly studied outcomes in mTBI are included in the table. Post Concussive Symptoms were determined by the Sports Concussion Assessment Tool (SCAT2-5), imPACT, Rivermead Post-Concussion Questionnaire (RPQ), Post-Concussion Symptom Inventory (PCSI), or Post-Concussion Symptom Scale (PCSS). Balance was determined by the Center of Pressure Displacement, Berg Balance Scale, Balance Error Scoring System, Vestibular-Ocular Motor Impairment. Mood symptoms were determined by the Beck Depression Inventory, Hamilton Rating Scale for Depression, PTSD Checklist, or Post-Traumatic Symptom Scale.

Cognitive function was assessed as an outcome in many DTI studies, as summarized in Table 3. Neuropsychological testing is used to assess overall cognitive function or performance on specific domains of function (e.g., psychomotor speed, working memory, etc.). Most studies found no significant association of either FA or MD with any measure of cognitive function. Out of 24 studies that examined the association of FA with general cognition, eight found lower FA associated with poorer cognitive function (76, 129, 161, 217, 239, 240, 242, 253), five studies found higher FA associated with poorer cognition (83, 204, 267, 277, 298), and 11 found no association (17, 38, 39, 43, 126, 131, 139, 177, 217, 220, 221). In all domains, more studies found higher FA was associated with better cognition (positive association—overall, attention, executive function, memory, motor, psychomotor speed, visuospatial function, IQ, and verbal fluence) as opposed to lower FA being associated with better cognition (negative association). However, across domains, many studies were not able to replicate these findings, demonstrating no significant association between FA and cognitive function. The association between higher FA and better cognitive performance was most notable among the executive function, IQ, and verbal fluency domains. Six out of 13 studies found better executive function associated with higher FA (127, 227, 233, 244, 246, 258). Of the remaining seven studies, one found the opposite association (229), and six found no association (17, 58, 127, 243, 249, 323). Among the five studies that investigated associations between IQ and FA, three found higher IQ associated with higher FA (145, 217, 240), while two found no association (271, 323). No studies demonstrated that higher IQ was associated with lower FA. Seven out of 13 studies found higher FA associated with better language fluency (133, 233, 240, 241, 253, 257, 323), while three studies demonstrated the opposite association (58, 129, 286), and three studies found no significant association (219, 248, 262). Fewer studies found significant associations between MD and cognitive function. In comparison to msTBI, many more studies attempted to find associations between DTI metrics and cognitive tasks. The overall trend is similar with higher FA associated with better cognitive performance across domains. In msTBI this was particularly evident for psychomotor speed, which was not replicated in mTBI.

**Table 3:** DTI associations with cognitive function.

DTI measure	Association	Overall cognition	Attention	Executive function	Memory	Motor	Psychomotor/Processing speed	Visuospatial	IQ	Verbal fluency/Language tasks/Reading fluency
FA	Positive *Poorer Performance-> Lower FA*	8 (76, 129, 161, 217, 239, 240, 242, 253)	3 (228, 243, 287)	6 (127, 227, 233, 244, 246, 258)	9 (75, 144, 145, 165, 244, 249, 253, 262, 330)	3 (76, 234, 329)	5 (22, 65, 127, 163, 257)	1 (58)	3 (145, 217, 240)	7 (133, 233, 240, 241, 253, 257, 323)
	Negative *Poorer Performance -> Higher FA*	5 (83, 204, 267, 277, 298)	2 (58, 130)	1 (229)	4 (129, 236, 248, 286)	1 (246)	4 (73, 131, 159, 203)			3 (58, 129, 286)
	None	11 (17, 38, 39, 43, 126, 131, 139, 177, 217, 220, 221)	3 (51, 249, 257)	6 (17, 58, 127, 243, 249, 323)	16 (17, 65, 130, 131, 163, 164, 230, 243, 252, 257, 271, 275, 282, 294, 323, 338)	4 (78, 126, 236, 252)	7 (17, 51, 67, 131, 164, 262, 323)	2 (65, 246)	2 (271, 323)	3 (219, 248, 262)
MD	Positive *Poorer Performance -> Lower MD*		1 (58)	1 (227)	2 (61, 65)	1 (246)				
	Negative *Poorer Performance -> Higher MD*	1 (161)		2 (246, 249)	2 (65, 252)	2 (78, 252)	1 (65)	2 (58, 65)		
	None	9 (17, 38, 39, 131, 139, 242, 277, 298, 338)	6 (61, 130, 221, 243, 249, 287)	6 (17, 58, 61, 243, 249, 323)	14 (17, 130, 131, 145, 163, 164, 230, 236, 243, 248, 253, 282, 294, 323)	2 (234, 236)	7 (17, 67, 73, 131, 163, 164, 323)	1 (246)	2 (145, 323)	6 (58, 133, 219, 248, 253, 323)

Studies included in this table attempted to find associations between DTI measures and cognition. Cognitive tasks are divided by domain. Positive association denotes higher FA or MD is associated with better cognition and negative association denotes higher FA or MD is associated with worse cognition.

Although some conclusions can be drawn from domains of clinical and cognitive functioning in which there have been many investigations, there is significant variability in study design and analysis that make drawing conclusions for many of the domains difficult. Within a domain of cognition, for example working memory, several tests across different studies may have been used to assess working memory. In addition, although these associations are all for participants with mTBI, there is considerable heterogeneity in populations across studies. These populations include studies of children and adults as well as a range of mechanisms and contexts of injury. While many studies investigated associations with outcomes in both the initial and current decade, studies in this decade often divided the mTBI group into subgroups based on clinical, cognitive, or imaging findings, which were therefore not included in Tables 2 or **3**, but similarly aimed to assess if changes in DTI were associated with specific clinical or cognitive symptoms. For example, Karlsen et al. divided the patients with mTBI into a group with post-concussion syndrome based on clinical symptoms and those without persistent symptoms and then compared DTI measures between these two groups (48).

### Limitations

This review must be considered in the light of several limitations. First, we have limited our search to English-language, peer-reviewed, original research articles. As such, the search did not encompass gray literature (conference papers, abstracts, etc.) and papers published in languages other than English. However, given the breadth of our search criteria, we believe that the studies included adequately capture the landscape of published literature on the use of DTI in mTBI over its second decade of reported use. In addition, our search covered mild through severe TBI, with this review focusing specifically on mTBI. We excluded studies exclusively investigating repetitive head impacts (RHI) associated with sub-concussive head injuries; a recent review addressed the use of DTI in RHI (347). As a consequence of classifying studies by TBI severity, those that did not specify TBI severity or included more than one severity without dividing the results accordingly were excluded from the review. Of the 553 original articles collected, only 11 did not report TBI severity; therefore, these exclusions are unlikely to bias our conclusions regarding mTBI literature over the past decade. Data extraction was conducted systematically to minimize errors. Each included study was extracted by a single reviewer and independently verified by a second reviewer; however, dual independent extractions were not completed for each study. Finally, the substantial heterogeneity across studies with respect to factors such as design, acquisition and analysis methods, and results reporting precluded a more quantitative analysis of the literature, such as a meta-analysis.

## Conclusion

Since the first decade of published studies on DTI and its applications to TBI were comprehensively reviewed, expansion and evolution of DTI applications to mTBI has continued. The body of scientific knowledge on DTI applications to mTBI has expanded in both quantity and scope, including notable increases in the proportions of larger and longitudinal studies, those employing whole brain and single subject analyses, and those addressing clinical outcomes or subgroups. The acquisition techniques and analysis methods have also migrated toward higher sensitivity methods, including higher magnet strength and lower slice thickness. The most salient feature of the study results remains that low FA is the most common finding identified in mTBI patients compared to controls, however the direction of the FA effect is more variable for mTBI compared to msTBI, where low FA was a much more consistent finding. Further standardization of reporting and methods for data harmonization that have become available hold potential for the pursuit of larger “meta-studies,” with potential to advance knowledge beyond the power of individual cohorts.

## Data Availability

The original contributions presented in the study are included in the article/supplementary material, further inquiries can be directed to the corresponding author.
